# Microplastics in Wastewater by Washing Polyester Fabrics

**DOI:** 10.3390/ma15072683

**Published:** 2022-04-06

**Authors:** Ana Šaravanja, Tanja Pušić, Tihana Dekanić

**Affiliations:** Department of Textile Chemistry and Ecology, Faculty of Textile Technology, University of Zagreb, Prilaz Baruna Filipovića 28a, HR-10000 Zagreb, Croatia; ana.saravanja@ttf.unizg.hr (A.Š.); tanja.pusic@ttf.unizg.hr (T.P.)

**Keywords:** microplastics, wastewater, textiles, polyester, polyester ageing

## Abstract

Microplastics have become one of the most serious environmental hazards today, raising fears that concentrations will continue to rise even further in the near future. Micro/nanoparticles are formed when plastic breaks down into tiny fragments due to mechanical or photochemical processes. Microplastics are everywhere, and they have a strong tendency to interact with the ecosystem, putting biogenic fauna and flora at risk. Polyester (PET) and polyamide (PA) are two of the most important synthetic fibres, accounting for about 60% of the total world fibre production. Synthetic fabrics are now widely used for clothing, carpets, and a variety of other products. During the manufacturing or cleaning process, synthetic textiles have the potential to release microplastics into the environment. The focus of this paper is to explore the main potential sources of microplastic pollution in the environment, providing an overview of washable polyester materials.

## 1. Introduction

Floating microplastics are the most widespread pollutant in the aquatic environment, acting as a contaminant for all aquatic organisms due to their constant deficiencies. Because of their small size, microplastics are consumed in large quantities by aquatic organisms, causing physiological problems. Microplastic ingestion has been related to lower food intake, developmental disorders, and behavioural changes. Data show that nearly 700 aquatic organisms worldwide are threatened by microplastic ingestion [1,2]. A variety of factors influences the biodegradation of microplastics, which is why it is important to understand the properties of the plastics. As plastics continue to disintegrate and defragment, the availability of microplastics will increase. Microplastics also have a tendency to change the density over time and, as a result, float because of biological fouling. The importance of microplastic composition has recently been highlighted in numerous publications. In general, a plastic composition refers to the polymer from which the plastic is made, and the density of microplastics is defined by this composition [1].

**Figure 1:**
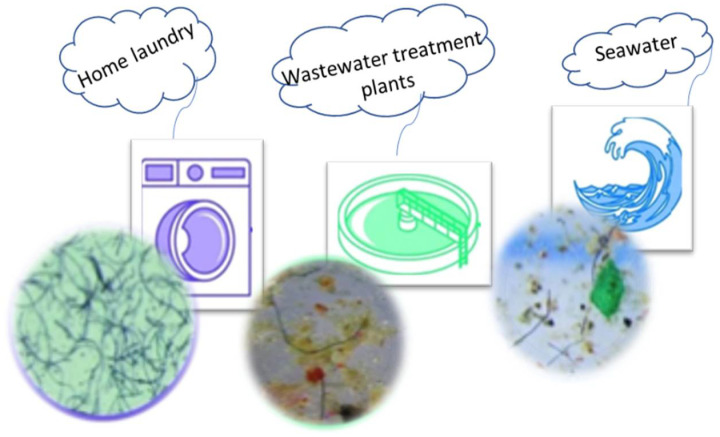
Microfibres detected in laundry effluent, wastewater and seawater [5].

All plastic products with a diameter less than 5 millimetres are classified as microplastics [3,4]. Two types of microplastics have been listed in the literature so far: the primary and secondary forms. The primary form of microplastics contains microgranules and can be found in cosmetics, whereas the secondary forms of microplastics are produced by the degradation process of larger plastic parts, e.g., poly(ethylene terephthalate) or PET bottles, or by the abrasion of synthetic textile materials, often called microfibers. However, there are certain inconsistencies in the use of the terms microplastics and microfibers, particularly in the context of textiles. As a result, these terms should not be misunderstood and used together [1,3]. Microplastics enter sewers, seas, and oceans due to human errors, carelessness, and fragmentation when using various materials such as paints, rubber, textiles or other plastics (Figure 1). 

By adding different types of additives to the polymer, they can be modified and their range of application extended. The most common additives are paints, fillers, UV protective agents, modifiers, lubricants, etc. Additives such as flame retardants and plasticizers have been found to be ubiquitous in various production processes and released into water and ground [6]. Regardless of technological advances and changing lifestyles, 2015 data shows that recycling remains a low priority. Only about 18% of PET is recycled, 10% of PE and high-density PE (HDPE), almost 6% of low-density PE (LLDPE), and less than 1% of PP [7].

Plastic additives, such as thermal stabilizers, are often used because they allow high processing temperatures. Plastic additives usually have negative connotations because there is insufficient evidence, as shown by the exposure to bisphenol A (BPA) in polycarbonate products. The EFSA (European Food Safety Authority) stated in 2007 that human exposure to BPA is below the TDI (lifetime exposure). Plasticizers are also found in microplastics, where there is a wide variety, including: adipates, phthalates, trimellitates, etc. [8]. 

## 2. Fragments of Plastics and Polymers 

The high productivity and extremely slow biotic breakdown of plastics cause them to spread in the environment as a result of adverse wastewater effects. Plastics that enter the aquatic environment and their residues may remain for months, or hundreds or thousands of years. During this time, they are defragmented by mechanical and photochemical processes, resulting in the formation of microplastics (<5 mm) or nanoplastics (<1 μm) [1,9,10]. It should be noted that the term polymer is often used in everyday language in a similar way to plastic, so it is necessary to clarify the basic difference. 

A polymer is a large molecule called a macromolecule, which consists of building units, so-called monomers. Macromolecules can be linear, branched, or crosslinked. The main difference between polymers and plastics is that plastics are mixtures/blends of two polymers or a polymer and low-molecular-weight compounds (additives) such as UV or thermal stabilizers, flame retardants, dyes, antioxidants, pigments, antimicrobial agents, lubricants, fillers, and others, according to the final applications [11,12,13,14]. 

Plastics can be used in many applications and various forms: clothing and textiles (PET, PAN, etc.), cookware (Teflon–PTFE), food containers (form HDPE, LDPE, or cups from PS), packaging (bags or bottles from PET), bearings (PA), epoxy glue, isolations (PS or PUR), silicone (heart valves), floor coverings (PVC), etc. [12,13,15,16,17]. Plastics based on PET, PE, PP and PA are mainly used in the textile industry.

Plastics are mostly derived from petroleum, including the category of polymers containing an ester functional group in each repeating unit of their main chain, but the most commonly used type of polyester in textile use is polyethylene terephthalate (PET), polypropylene, polyethylene, and polyvinyl chloride, of which polypropylene and polyethylene are common and standard products, Table 1.

Low-density plastics, such as PP and PE, form fragments with a lower density than water and thus float on the water, whereas higher-density polymers, such as PET, form plastics that do not float but already settle depending on their movement and sinking speed. Positively charged plastics usually float on the water surface, but only for a short time prior to becoming contaminated and mixed in with other waste. 

One of the most important properties of plastic is its durability. Due to inadequate waste management, plastic pollution occurs on land and in the sea. Larger pieces of plastic are scattered, which is particularly visible in coastal areas where the effects of waves and UV light are strongest. High temperatures combined with intense UV radiation affect the decomposition of plastic, whereas lower temperatures and less UV radiation result in much slower decomposition [19]. Depending on the causes of decomposition, there are several mechanisms: biodegradation, photodegradation, thermal degradation, thermo-oxidative degradation, and hydrolysis [18].

Photodegradation, photo-oxidation, UV decomposition, and oxidative degradation are synonyms for the same process. It is a phenomenon in which UV light in combination with atmospheric oxygen changes the physical and chemical structure of plastics [20]. The rate of degradation can vary depending on the environment and temperature, but photodegradation has been shown to be the most influential factor in the degradation processes of this type [20,21]. UV light affects plastics depending on the conditions present [22]. Ranjan and Goel have shown that the degree of photodegradation varies depending on the environment. The oxygen content of water and oxygen are needed for the degradation process [23].

Biodegradation is carried out by microorganisms under aerobic and anaerobic conditions [24,25]. Microorganisms change the chemical structures, sizes, shapes, and masses of plastics through hydrolysis. Aerobic degradation is a process that uses oxygen as an oxidant and decomposes organic matter to carbon dioxide and water. This process often takes place in nature where oxygen is abundant. Anaerobic decomposition is the process of decomposition in the absence of oxygen. Thermal degradation refers to chemical changes in polymers as a result of elevated temperature [26]. Thermooxidative degradation is a slow oxidative process at moderate temperatures, and hydrolysis means degradation caused by water reaction [18].

Direct plastic contamination is an irreversible process because plastic is not degradable and there is almost no way to collect these microplastic particles in water resources after the plastic has been sprayed into microplastics. A real example of a primary plastic product where plastic directly plays an important role in cleansing the face or skin, besides toothpaste, is exfoliating creams. Here, plastic particles, usually polyethylene (PE) particles, remove dirt from the face. After rinsing the face or body, these particles end up in wastewater and thus in the sewage system. As a result, the United States has banned the use of plastic beads in creams and cosmetics. This has led some industries to list and identify sustainable particles that are certainly biodegradable and are almost equivalent to the microplastic particles found in cosmetics, even in terms of price, solvent resistance, surface shape, uniform size, and mechanical properties. Biodegradable products have been replaced by: natural hard material (human walnut), synthesized bio-based polymers (polylactic acid), and natural polymers (starch, lignin) [27]. There is a growing interest in the development of green plastic using natural resources. Green plastic is divided into two groups: green plastic made from monomers derived from biomass such as vegetable oil, and natural polymers such as chitin or lignin, which have extremely high biocompatibility and biodegradability [13].

### Ageing of Polymers

Ageing is a term used in polymer science when the properties of polymers change over a period of time. Previous studies have shown that ageing causes the degradation of polyester at the molecular level. Theoretically, there are two types of ageing mechanisms: physical and chemical. Physical ageing is the most common type of ageing, corresponds to changes that have occurred in the composition (water absorption) and in the chain configuration itself, most often when the intermediate molecules have been modified but without changing the chemical structure of the polymer. The main groups of physical ageing are structural relaxation or a reduction in free volume and physical ageing, which occurs due to solvent penetration or release of plasticizer. Chemical ageing, on the other hand, is the result of reactions with external agents such as water, oxygen, UV radiation, or ionizing radiation. Considering the problem of polyester during washing, the emphasis is on the interaction of polyester with water. Physical ageing in amorphous parts of polymers occurs below the glass transition temperature, *Tg*. Penetration of water or other small molecules into the interior of the polymer leads to a sharp drop in the glass transition temperature. Water is a polar solvent, and its solubility in polyester is expected to be very low. Water has the ability to react with ester groups, so in this case, it is a type of physical ageing with water. It is a reversible process that is often accompanied by irreversible damage that occurs in the fibre matrix itself or at the fibre interface [28,29,30,31]. 

**Figure 2:**
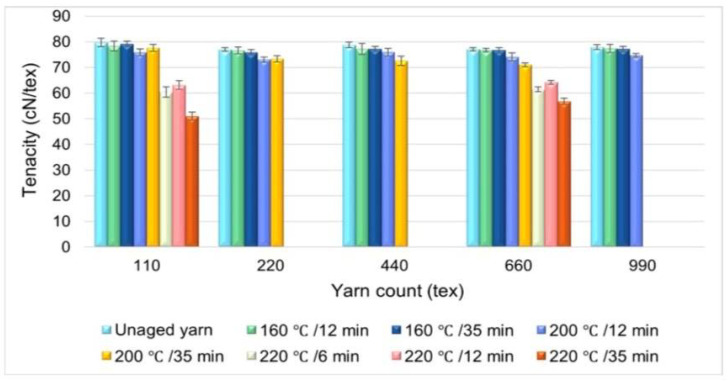
The relationship between the ageing time at ageing temperatures of 160, 200, and 220 °C and yarn strength [32].

One of the most important mechanisms for degradation is physical ageing due water or wet ageing. Lemmi et al. studied the nature of physical ageing of polycrystalline PET, varying the temperature and duration of ageing, and then compared how ageing directly affects fibre strength (Figure 2) [32].

The results obtained show that the materials aged for 12 and 35 min at a temperature lower than 160 °C show no difference from the untreated yarns. Materials processed above 160 °C but less than 200 °C show a decrease in strength below 1.11 cN/tex. Polyester samples aged at temperatures of 140 °C and 160 °C for a period of 12 min and 35 min showed almost no difference in strength compared to the untreated samples. However, the strength decreased with increasing temperature, and the toughness also decreased by about 5% at 200 °C and 19% at 220 °C. A sample aged at 220 °C for 35 min has almost 31% lower strength than the untreated polyester.

The parameters of thermal ageing significantly affect the mechanical and physical properties of the samples. In summary, the strength and thermal elongation are inversely proportional to the ageing temperature [32].

## 3. Textiles—Source of Microplastic Pollution 

Textiles are also considered one of the major sources of microplastic pollution. Microplastics from textiles generally have a fibre shape, which is why they are often referred to as microfibres [33,34]. The main sources of microplastic fibres released from textiles are textile manufacturing and industrial and household washing. Cai et al. have shown that, during yarn spinning, microplastic fibres are released five times more from textiles with processed surfaces, such as fleece or plain brushed. Additionally, many studies have provided estimates of microfiber emissions from synthetic textiles during machine washing. Numerous studies and results show that textiles are among the main potential polluters due to the release of fibres, and that one of the reasons for this is the washing process [34,35,36,37,38,39,40,41,42,43,44,45,46,47,48,49,50]. Research has shown that the type of construction itself affects the amount of microfibers released. It would be convenient to use fabrics with a compact weave and as high a density as possible. The first wash releases most of the microfibers, and as the number of washes increases, the release will decrease. It is emphasized that washing parameters such as time and temperature have an influence on the release of microfibers [44]. Some possibilities to avoid this phenomenon are listed in Table 2.

It has been shown that, among all textile fibres, polyester and cotton are the most widely used, with a total annual demand of 46 million tons, if polyester is included [1,35,52,53].

Polyester fibres belong to a group of fibres composed of macromolecules, synthesized polymers with a linear structure characterized by ester bonds (-CO-O-) linking the constitutional units, after which the whole group is named. Poly(ethylene terephthalate), PET, is the most abundant polymer in the polyester fibre group. Taking into account the data from 2017, PET accounts for 50% of the total man-made fibres produced, and 14% are made from recycled PET [1]. 

As for its properties, polyester has a density of 1.37–1.45 g/cm^−3^, sinks very quickly, is non-biodegradable, and shows some weathering resistance. Although polyester is resistant to weathering, the fragmentation mechanisms are not, so photo-oxidation and hydrolysis can occur in marine environments. The change in pH in the ocean can change the chemical equilibrium of the microplastic itself by increasing or decreasing the rate of chemical leaching from the polyester surface, i.e., PET, which is generally considered a safe fibre, may become extremely hazardous in the near future [1,54,55]. 

Recently, PET polymeric materials and PET fibres, especially in the form of plastic bottles and packaging, have often raised environmental issues related to microplastics. It should be noted that polyester is not harmful to health and the environment, but due to its high presence, i.e., its high volume in waste, low biodegradability, and partial resistance to biological and atmospheric agents, the biggest problem is the release of microplastics into the environment. Due to these characteristics, polyester belongs to the category of environmentally unfriendly materials. Increasing the percentage of recycled PET production is economically and environmentally acceptable due to two segments:

- Creation of added value with low quality materials;
- Reduction in the plastic waste increase [56].

Among the studies reviewed, polyester is the most commonly investigated textile material because of its widespread use in the textile industry [53]. Some other studies deal with other textile materials such as polyamide [57,58,59,60,61], polyacrylic [62], and their blends [63,64]. It has also been found that natural fibres such as cotton minimise the risk of environmental pollution compared to synthetic textiles due to its biodegradability [57,58].

### 3.1. Textiles—The Release in the Washing Process

**Figure 3:**
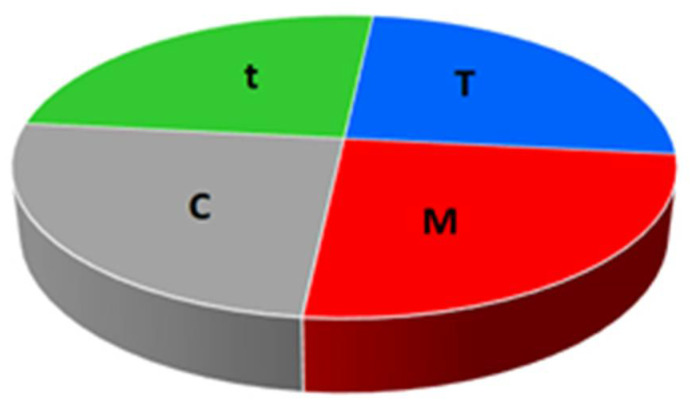
Washing factors according to Sinner’s cycle: t—time, T—temperature, M—mechanics, C—chemistry.

It is well-known that, in the washing process, five partners act in synergy: textile material, water, detergent, washing machine and stain. Water serves as a medium for the transport of thermal and mechanical energy, as well as for washing. Various physical and chemical mechanisms occur during textile washing. The main parameters that affect washing performance are mechanics, temperature, chemistry, and time [65,66,67,68,69]. Their joint action is responsible for a satisfactory washing effect (Figure 3).

Washing in household washing machines increases the mechanical agitation (action), which shortens the time, and the factors in the Sinner’s cycle are changed. As far as chemicals are concerned, this group includes detergents that are biodegradable and, therefore, environmentally friendly. 

The washing of household textiles has been shown to be an important source of microplastic contamination, although the release of fibres from textiles is not yet fully understood [57]. In some literature sources, the washing process is mentioned as a source of contamination of plastic fibres. These data showed that a single garment can release more than 1900 fibres per wash cycle, and that almost all garments emit more than 100 fibres per litre of wastewater [65]. In addition, studies have shown a release of 0.033–0.039% by weight of fibres from polyester garments after washing, although it should be noted that no experimental conditions are given. In conclusion, home textile washing is a continuous source of microplastic emissions in wastewater, and the relationship between the process of washing, and the release of microplastics is proportional.

As for the release of microplastics during the washing process, it should be noted that this problem applies to a greater extent to synthetic textiles, not so much to natural fibre materials, since natural fibres are biodegradable. The representative of the synthetic fibres is usually the polyester fibre. According to the available literature data, the release of fibres during washing is most often studied at different temperatures, with the addition of different amounts of detergents, and using certain mechanics and time. The results showed that the average size of the released fibre was 11.9 to 17.7 μm in diameter, and the length was 5.0 to 7.8 mm [36,37,62]. 

Kelly et al. [70] have shown the relationship between the effect of time and washing temperature, confirming that at smaller temperature and time intervals there is no difference, that at 15 and 30 °C for 15 to 30 min there is no effect on the release of microplastics, whereas at higher temperatures from 60 °C, there is a significant increase in the release of microplastics when polyester fabrics are processed. When mentioning and comparing temperatures of 15 and 30 °C, it should be noted that these temperatures fall into the category of “cold” washing. When comparing temperatures, there is a significant increase in the amount of microplastics when washing at 60 °C, but also at less than 60, whereas below 30 °C, there is only a minimal change in the release of microplastics. Therefore, the temperature is not such an important factor affecting the increase in or release of microplastics. Considering the mechanics of washing itself due to a prolonged time cycle of washing, the research found that the release of microplastic would be significantly increased, although it should be noted that there was no significant difference in the release of microplastics at a duration of 8 h. Additionally, the detergent has no effect on the release of microplastics. However, there are some doubts about the effect of detergents. For example, the study shows that steel balls are used with the detergent, which could interact with the detergent as a mechanic when they would enhance the effect of the detergent, thus forcing it into the fabric itself and creating many bubbles. However, this research has not yet been sufficiently explored, but I would like to draw attention to the fact that the safe type of the child itself has some influence on the release of the microplastics [70].

Although there are many publications on the release of microfibres during washing, other possible pathways of microplastic particle release into the environment have only recently been investigated. One of these is drying in tumble dryers [71,72,73]. Household tumble dryers play an important role in the release of microfibres from textiles into the environment or into the air. This is because the air from the dryer is usually not treated before it is released into the environment, and the microfibres are released directly from the dryer or into the surrounding space or through the ventilation pipe into the environment [71,72,73,74]. As the textiles spin in the dryer, the microfibres could be released from the textiles, especially at higher temperatures. The release of microfibres from large commercial dryers is not known but could also be significant and not negligible. Furthermore, if the dryers are not connected to the ventilation system, human microfibres can be released directly from the air of the enclosed space [73,75]. Microplastics have been detected in indoor and outdoor air all over the world. Based on a normal exposure scenario, it has been estimated that a child could ingest more than 900 microplastic particles per year via dust [76].

When investigating the effect of repeated washing cycles on the release of microplastic particles, Sillanpää et al. concluded that the release was reduced by about 90% in the last cycles [35]. On the other hand, Hartline et al. found that older garments released more microplastics during a continuous wash cycle of several hours. Various observations were also made when investigating the effect of detergents on the release of microplastics. For example, Napper and Thompson and De Falco found that the presence of detergents generally leads to an increased release of microplastic particles. Pierc and his colleagues reported that the detergent had no significant effect on the release of microplastics [35,61,62,70,74,77].

The release of textile fibres may also be affected by parameters other than the type of the fabric and the washing and drying process. Some other studies have investigated the effects of water hardness, water softeners, temperature, and the type of washing machine [39,59,61,62,74,77,78,79]. Zambrano et al. reported that the type of shedding is affected by fibre friction, shape, thickness, stiffness, and abrasion resistance. They linked the stronger shedding of cotton fibres mainly to the stronger hairiness of cotton fabric compared to other fibres studied. The authors found that polyester is one of the materials with the lowest abrasion resistance compared to cellulose fibres. They concluded that fabrics with higher abrasion resistance, higher yarn strength, and lower hairiness are desirable factors to reduce fibre shedding during washing [78]. 

Among the synthetic materials used, polyester fibres shed more microfibres compared to other synthetic fibres such as acrylic and polyamide. Knitted fabrics were also found to release more fibres. Other researchers suggest that the selection of a suitable spinning method could control the structural compactness and thus solve the problem of loose structures, hairiness, and twisting of the yarn [43,80]. De Falco et al. have found that fabrics release more microfibres than knitwear, depending on the type of fibres used in the textile production. Knitwear is made of filaments, whereas woven fabric is made of double yarn with greater hairiness. As for the surface mass of the textiles, they have shown that the release of fibres is not affected [77].

**Figure 4:**
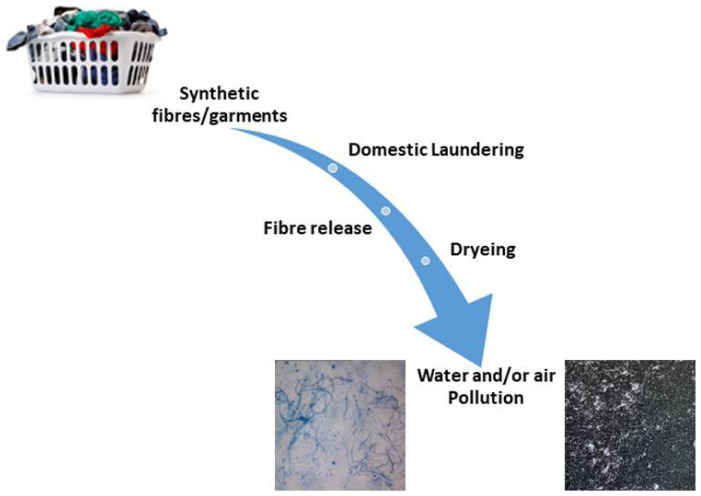
The microfiber shedding mode.

Further research results show that the number of microfibers released from polyester and cotton fabrics after the first wash varied from 2.1 × 105 to 1.3 × 107 and that the largest number of fibres came from cotton fabrics. Indeed, the results showed that the annual emission of microfibers in Finland was estimated to be 154,000 kg (PES) and 411,000 kg (cotton) [35]. Synthetic waste effluents from washing machines contain released fibres which are then transported to the wastewater and sewage system, and are deposited or floated in them (Figure 4). 

Guo et al. found that polyester microfibers are able to change soil structure and organic matter transfer, which also shows that they can affect not only water resources but also soil function. They wanted to show what effect microfibers have on enzymes by storing soil organic carbon, the micromolecules. They hypothesized that polyester microfibers have a great tendency to alter the overall microbial community in the soil as well as the activation of enzymes and the effect of cycling organic carbon in the soil. The authors concluded that the addition of polyester microfibers had the same effect on the whole soil community, and the activity of lactose and cellulose in the soil was also improved by the addition of microfibers, but the addition of microfibers also reduced the total amount of organic matter in the soil [81].

### 3.2. Wastewater and Microplastics

Wastewater treatment plants can serve as an entry point for microplastics into the environment. The water treatment process is often influenced by the particle size. Large solid particles are removed from wastewater treatment plants, and thus the concentration of organic matter is reduced [4]. To reduce the amount of particles in wastewater, some wastewater treatment plants use a filter or a membrane bioreactor (MBR). Pretreatment is also a required step in wastewater treatment plants, which is divided into three stages, primary, secondary, and tertiary [6], each involving different procedures:

- mechanical;
- chemical;
- physico-chemical;
- biological; and
- chemico-biological.

Over the past decade, wastewater treatment has been constantly needed to improve wastewater quality. However, the final technologies to improve wastewater quality are not specifically designed to remove microplastics. According to the literature review, new wastewater treatment methods may improve the final step of removing microplastics from wastewater [82]. These newly developed technologies can effectively remove microplastics from wastewater, but they are a very expensive process, which is difficult to install into existing plants and are used only for applications that require high quality standards An example of this is membrane bioreactors, which adsorb only water and small particles after primary and secondary treatment using cross-flow filtration. Another limitation of this technology is the high demand for energy sources, which results in a high cost for the process, making it uneconomical [4].

Thanks to rapid sand filters and discfilters, scientists have achieved the fast removal of microplastics by physical separation [83]. Microplastic can also be isolated during filtration, microfiltration 0.1–1 μm, ultrafiltration 2–100 μm, and nanofiltration 2 nm. Biochar has been used in sand filter systems for filtration efficiency. Microplastic particles with a diameter of up to 10 μm can be removed with an efficiency of 95% [84]. Electrocoagulation, in which coagulants are produced by electric current, is also applicable. In most cases, this involves iron or aluminium electrodes reacting with hydroxide ions formed after electrolysis to form hydroxide coagulants. The microplastic becomes very unstable in the presence of the coagulants formed and remains suspended in the sludge blanket. The results showed that about 90% of microfibers were removed [84] by electrocoagulation.

The number of microplastics can be influenced by lifestyle, population, climate, and seasonal conditions. The fact is that the amount of microplastic is highest in warmer conditions than in colder ones. It has been shown that wastewater samples contain fragments of PP and PE, whereas sludge contains PA, PET, and PS, as denser polymers are deposited during the treatment process [85]. Studies have also shown that a human can ingest between 39,000–520,000 microplastic per year through food and beverages, with levels naturally depending on age and gender [86]. Thanks to the method, it is possible to distinguish microplastics from non-plastic particles on the filter membrane using μ-FTIR [87].

## 4. Mitigation Measures for Microplastic Contamination

In this context, synthetic textiles stand out and disintegrate, and some measures are proposed to be applied during the production process. 

### 4.1. Prevention

Adjusting the next steps has a significant impact on preventing the formation of microfibers. First of all, it is recommended to use high-quality products, focusing on clothes made of natural fibres. Then, if possible, it is important to avoid the use of mechanically finished fabrics such as fur or fleece. Furthermore, it is recommended to use appropriate chemicals (less alkali detergents—liquid or gel, softener, and other finishing agents) that reduce the release of microfibers. The emphasis is that the main problems include microplastics/microfibres and insufficient regulatory standards [88]. Airing out clothes after each use also reduces the number of washes and thus the release of microfibers [60]. 

### 4.2. Measures Proposed for The Production Process

The impacting parameters are: twisting yarn to increase elasticity and resistance, reducing the thickness because this automatically reduces the number of fibres, increasing the fabric density because this increases the strength of the structure, reducing the use of short fibres, and careful choice of textile auxiliaries to prevent friction between fibre–detergent and fibre–fibre, as well as to protect against mechanical stress in the washing machine [43,78,80,89].

The study mentions two biodegradable coatings as protection against physical stress, namely chitosan and pectin. Textile Company Lenzing AG announced that it uses alternatives to biodegradable materials such as Tencel (Lyocel), viscose, and Modal. The functionalization of the protective layer itself is based on the use of pectin, a natural polysaccharide that is extremely cheap and very readily available. It is known as a waste product of sugar and sunflower oil production. The test results of the washed treated fabrics showed a significant reduction in microfibers by up to 90% compared to untreated fabrics and a promising resistance to the washing process as well [90].

The proposed measures cover the exclusive use of an ultrasonic cutter to reduce the amount of precipitated fibres by about 50%, less brushing during processing, the use of recycled PET, and the removal of existing fibres before shipment.

Innovative surface treatment aimed to create a protective layer on the surface to reduce the release of microfibers from the surface. Chitosan is an amorphous solid white, harmless, odourless, and biodegradable biopolymer. It is soluble in acid but not soluble in alkali, water, or any organic solvent. The production of chitosan lacks the desired purity, which is extremely important in the pharmaceutical industry. The chitosan used in the textile industry is of low purity and can be used directly in fibre production. Studies have shown that chitosan can reduce the harmful release of microplastics during the dyeing process [91,92]. Chitosan also has an antibacterial effect and is used in finishing to obtain antibacterial fibres [93,94,95,96,97]. Some studies have shown good resistance to washing and antibacterial activity of chitosan-treated fabrics against *Staphylococcus aureus* in up to five washing cycles [93,94]. The treatment of polyester fabrics with chitosan leads to a significant antistatic effect in addition to its antibacterial properties. Matsukawa et al. treated polyester fabrics with chitosan by hydrolysing its surface with a sodium hydroxide solution to incorporate it into a functional group (-COOH) [23,26]. It is also important to know that polyester can be dyed directly if it has been previously treated with chitosan in an alkaline treatment and impregnated in this bath and is then ready for dyeing. The alkaline treatment improves the adhesion of chitosan to the polyester surface, resulting in a stronger depth of colour [98,99]. It is noted that obtaining better effects depends on the amount of chitosan and its properties, such as the degree of deacetylation or molecular weight. However, the amount of chitosan should be carefully dosed because the higher the viscosity, the more undesirable the effects.

### 4.3. Mechanical Means of Preventing the Release of Fibres during Use

*Cora Ball* is made of soft plastic, with pores that can adhere microfibers to its surface in the washing process. Collected microfibers can easily be removed by hand. The results show that the total amount of fibres absorbed by the ball is about 35% in each wash. It is also important that the used ball retains the ability to collect microfibers compared to a newly purchased ball [89]. 

### 4.4. Innovative Methods for the Direct Removal of Microparticles from the Aquatic Environment

It is possible for microplastics to degrade through the action of microorganisms that degrade microplastic to biomass, carbon dioxide, methane, water, and other inorganic compounds. Degradation parameters such as environmental parameters, UV radiation, solar radiation, moisture, and the physical and chemical properties of the polymer play an important role. In addition to microorganisms, microplastics can also be degraded by bacteria. Biocatalyst cells, *Comamonas Testosteroni*, are able to degrade PES and thus reduce the release of microplastics. At the beginning of the process, the diameter of PES was 7.30 μm; after treatment in alkaline medium, the diameter decreased to 1.58 μm, which promoted the rapid degradation of PES under alkaline degradation conditions by bacteria, and by biosorption, which allowed the adsorbate to bind to the surface of the adsorbent. Studies have shown that algae and brown algae tend to adsorb microplastics due to the presence of alginic acid in their walls. The functional carboxyl group is present in brown algae, and thanks to this group, the plastic can bind to the adsorbent [100,101]. 

## 5. Conclusions

Microplastics have become a serious threat to the environment and human health. Therefore, the gradual release and use of microplastics, which occurs directly in the fibre production itself, must be drastically reduced and be part of a global initiative, even before research studies on the long-term risks and consequences are available. The environmental aspects of microplastics are not sufficiently considered, it is not given enough importance, and most people are not aware of its negative effects. There is a need to develop as many programs as possible to monitor microplastics and thus play a key role in the prevention and management of microplastic pollution. Many countries have not developed a strategic approach to the largest sources of microplastics accumulating in water, nor have they developed processes to clean them up efficiently. There are several research projects that have investigated the impact of microplastics on final waste, as well as the removal of microplastics during each step of the process in wastewater treatment plants. Therefore, it is crucial that the machines are economical, energy efficient, and environmentally friendly to reduce the emission of microplastics as cost-effectively as possible. As far as polyester and the release of microplastics are concerned, the primary objective is to produce polyester with optimal structural parameters and switch to the most recycled production possible. It is necessary to use the most suitable agents that protect the structure of the polymer and prevent the release of microplastics during washing. It is also necessary to optimize the washing and drying processes of synthetic materials, as they are unique and market relevant. Finally, it is obvious that this interdisciplinary topic will continue to be relevant in the future and there is great potential for further research.

## Figures and Tables

**Table 1:** Applications and specific density of different synthetic polymers found in the marine environment [9,18].

Categories	Common Applications	Specific Density [g/cm^3^]
Polyethylene (PE-LDPE, LLDPE)	Plastic bags, six-pack rings, bottles	0.91–0.93
Polypropylene (PP)	Rope, bottle caps, netting	0.90–0.92
Foamed polystyrene (PS)	Cups, buoy	0.01–1.05
Polystyrene (PS)	Plastic utensils, food containers, packaging	1.04–1.09
Polyvinyl chloride (PVC)	Bags, tubes	1.16–1.30
Polyamide or nylon (PA)	Ropes	1.13–1.15
Polyethylene terephthalate (PET)	Beverage bottles	1.34–1.39
Polyester resin + fibreglass	Textiles	>1.35
Polycarbonate (PC)	Electronic compounds	1.20–1.22
Cellulose acetate (CA)	Filter cigarettes	1.22–1.24
Polytetrafluoroethylene (PTFE)	Teflon items, tubes	2.10–2.30

**Table 2:** Possibility of preventing the release of microfibers [51].

**Textile parameters**	Type of fibres	Hydrophilic fibres release more fibres than synthetic ones The strength can also affect tearing
	Yarn properties	Yarns with more twists and longer filaments release less microfibres
	Structure of fabric	Thermally cut fabrics release less than mechanically cut fabrics The influence of knitted and woven structure is not entirely clear
	Ageing of fabric	The impact is not predictable because the garment does not pass a complete lifecycle
**External parameters**	Washing machine	Vertical drum machines contribute more to the release than horizontal ones, although this is related to the bath ratio

## Data Availability

No new data were created or analyzed in this study. Data sharing is not applicable to this article.

## References

[1] Issac M.N., Balasubramanian K. (2021). Effect of microplastics in water and aquatic systems. Environ. Sci. Pollut. Res..

[2] Marn N., Jusup M., Kooijman S., Klanjscek T. (2020). Quantifying impacts of plastic debris on marine wildlife identifies ecological breakpoints. Ecol. Lett..

[3] Kolbe S. (2018). Microplastics or microfibers?—Conceptual confusion, Research Institute for Textiles and Clothing (FTB), Niederrhein University of Applied Sciences, Moenchengladbach, Germany. Tekstil.

[4] Westphalen H., Abdelrasou A., Glavan M. (2018). Challenges and treatment of microplastics in water. Water Challenges of an Urbanizing World.

[5] Vassilenko K., Watkins M., Chastain S., Posacka A., Ross P.S. Me, My Clothes and the Ocean: The Role of Textiles in Microfiber Pollution, Science Feature. Ocean Wise Conservation Association, Vancouver, QC, Canada, 16p. https://assets.ctfassets.net/fsquhe7zbn68/4MQ9y89yx4KeyHv9Svynyq/8434de64585e9d2cfbcd3c46627c7a4a/Research_MicrofibersReport_191004-e.pdf.

[6] Carr S.A., Liu J., Tesoro A.G. (2016). Transport and fate of microplastic particles in wastewater treatment plants. Water Res..

[7] Thiounn T., Rhett C.S. (2020). Advances and approaches for chemical recycling of plastic waste. J. Polym. Sci..

[8] Andrady A.L., Neal M.A. (2009). Applications and societal benefits of plastics, Philosophical transactions of the Royal Society of London. Series B. Biol. Sci..

[9] Espinosa C., Esteban M.Á., Cuesta A., Larramendy M.L., Soloneski S. (2016). Microplastics in aquatic environments and their toxicological implications for fish. Toxicology—New Aspects to This Scientific Conundrum.

[10] European Parliament’s Policy Department for Citizens’ Rights and Constitutional Affairs (2020). The Environmental Impacts of Plastics and Micro-Plastics Use, Waste and Pollution: EU and National Measures—Study. https://www.europarl.europa.eu/RegData/etudes/STUD/2020/658279/IPOL_STU(2020)658279_EN.pdf.

[11] Čatić I., Barić G., Cvjetičanin N., Galić K., Godec D., Grancarić A.M., Katavić I., Kovačić T., Raos P., Rogić A. (2020). Polimeri—Od prapočetka do plastike i elastomera. Polimeri.

[12] Van der Vegt A.K. (2002). From Polymers to Plastics.

[13] Sazali N., Ibrahim H., Jamaludin A.S., Mohamed M.A., Salleh W.N.W., Abidin M.N.Z. (2020). A short review on polymeric materials concerning degradable polymers. IOP Conf. Ser. Mater. Sci. Eng..

[14] Material Properties of Plastics. https://application.wiley-vch.de/books/sample/3527409726_c01.pdf.

[15] Nmazai H. (2017). Polymers in our daily life. BioImpacts.

[16] Maddah H.A. (2016). Polypropylene as a promising plastic: A review. Am. J. Polym. Sci..

[17] Eyerer P., Eyerer P. (2010). Plastics: Classification, characterization, and economic data. Polymers—Opportunities and Risks I. The Handbook of Environmental Chemistry.

[18] Andrady A.L. (2011). Microplastics in the marine environment. Mar. Pollut. Bull..

[19] Andrady A.L., Bergmann M., Gutow L., Klages M. (2015). Persistence of plastic litter in the oceans. Marine Anthropogenic Litter.

[20] Yousif E., Haddad R. (2013). Photodegradation and photostabilization of polymers, especially polystyrene: Review. SpringerPlus.

[21] Lambert S., Sinclair C.J., Bradley E.L., Boxall A.B.A. (2013). Effects of environmental conditions on latex degradation in aquatic systems. Sci. Total Environ..

[22] Julienne F., Delorme N., Lagarde F. (2019). From macroplastics to microplastics: Role of water in the fragmentation of polyethylene. Chemosphere.

[23] Ranjan V.P., Goel S. (2019). Degradation of Low-Density Polyethylene Film Exposed to UV Radiation in Four Environments. J. Hazard. Toxic Radioact. Waste.

[24] Gu J.D. (2003). Microbiological deterioration and degradation of synthetic polymeric materials: Recent research advances. Int. Biodeterior. Biodegrad..

[25] Hammer J., Kraak MH S., Parsons J.R. (2012). Plastics in the Marine Environment: The Dark Side of a Modern Gift. Rev. Environ. Contam. Toxicol..

[26] Faravelli T., Pinciroli M., Pisano F., Bozzano G., Dente M., Ranzi E. (2001). Thermal degradation of polystyrene. J. Anal. Appl. Pyrolysis.

[27] Ju S., Shin G., Lee M., Koo J.M., Jeon H., Ok Y.S., Hwang D.S., Hwang S.Y., Oh D.X., Park J. (2021). Biodegradable chito-beads replacing non-biodegradable microplastics for cosmetics. Green Chem..

[28] Hunter L.W., White J.W., Cohen P.H., Biermann P.J. (2000). A materials aging problem in theory and practice. Johns Hopkins APL Tech. Dig..

[29] Richaud E., Verdu J. (2019). Aging behavior and modeling studies of unsaturated polyester resin and unsaturated polyester resin-based blends. Unsaturated Polyester Resins: Fundamentals, Design, Fabrication, and Applications.

[30] Salopek Čubrić I., Čubrić G., Potočić Matković V.M. (2021). Behavior of Polymer Materials Exposed to Aging in the Swimming Pool: Focus on Properties That Assure Comfort and Durability. Polymers.

[31] White J.R. (2006). Polymer ageing: Physics, chemistry or engineering? Time to reflect. Comptes Rendus Chim..

[32] Lemmi T.S., Barburski M., Kabziński A., Frukacz K. (2021). Effect of thermal aging on the mechanical properties of high tenacity polyester yarn. Materials.

[33] Roos S., Levenstam Arturin O., Hanning A.-C. (2017). Microplastics Shedding from Polyester Fabrics, Mistra Future Fashion Report No 2017:1, SEREA. http://mistrafuturefashion.com/wp-content/uploads/2017/06/MFF-Report-Microplastics.pdf.

[34] Manshoven S., Smeets A., Malarciuc C., Tenhunen A., Mortensen L.F. Microplastic Pollution from Textile Consumption in Europe, Eionet Report—ETC/CE 2022/1. https://www.eionet.europa.eu/etcs/etc-ce/products/etc-ce-products/etc-ce-report-1-2022-microplastic-pollution-from-textile-consumption-in-europe.

[35] Sillanpää M., Sainio P. (2017). Release of polyester and cotton fibers from textiles in machine washings. Environ. Sci. Pollut. Res..

[36] De Falco F., Cocca M.C., Avella M., Thompson R.C. (2020). Microfiber Release to Water, Via Laundering, and to Air, via Everyday Use: A Comparison between Polyester Clothing with Differing Textile Parameters. Environ. Sci. Technol..

[37] Galvão A., Aleixo M., De Pablo H., Lopes C., Raimundo J. (2020). Microplastics in wastewater: Microfiber emissions from common household laundry. Environ. Sci. Pollut. Res..

[38] Gaylarde C., Baptista-Neto J.A., da Fonseca E.M. (2021). Plastic microfibre pollution: How important is clothes’ laundering?. Heliyon.

[39] Hernandez E., Nowack B., Mitrano D. (2017). Polyester Textiles as a Source of Microplastics from Households: A Mechanistic Study to Understand Microfiber Release During Washing. Environ. Sci. Technol..

[40] Cai Y., Yang T., Mitrano D.M., Heuberger M., Hufenus R., Nowack B. (2020). Systematic Study of Microplastic Fiber Release from 12 Different Polyester Textiles during Washing. Environ. Sci. Technol..

[41] De Falco F., Di Pace E., Cocca M., Avella M. (2019). The contribution of washing processes of synthetic clothes to microplastic pollution. Sci. Rep..

[42] Volgare M., De Falco F., Avolio R., Castaldo R., Errico M.E., Gentile G., Ambrogi V., Cocca M. (2021). Washing load influences the microplastic release from polyester fabrics by affecting wettability and mechanical stress. Sci. Rep..

[43] Rathinamoorthy R., Balasaraswathi S.R. (2021). A review of the current status of microfiber pollution research in textiles. Int. J. Cloth. Sci. Technol..

[44] Choi S., Kwon M., Park M.-J., Kim J. (2021). Characterization of Microplastics Released Based on Polyester Fabric Construction during Washing and Drying. Polymers.

[45] Čurlin M., Pušić T., Vojnović B., Dimitrov N. (2021). Particle Characterization of Washing Process Effluents by Laser Diffraction Technique. Materials.

[46] Schöpel B., Stamminger R. (2019). A Comprehensive Literature Study on Microfibres from Washing Machines. Tenside Surfactants Deterg..

[47] Henry B., Laitala K., Klepp I.G. (2019). Microfibres from apparel and home textiles: Prospects for including microplastics in environmental sustainability assessment. Sci. Total Environ..

[48] Bayo J., Ramos B., López-Castellanos J., Rojo D., Olmos S. (2022). Lack of Evidence for Microplastic Contamination from Water-Soluble Detergent Capsules. Microplastics.

[49] Haap J., Classen E., Beringer J., Mecheels S., Gutmann J.S. (2019). Microplastic Fibers Released by Textile Laundry: A New Analytical Approach for the Determination of Fibers in Effluents. Water.

[50] Luogo B.D.P., Salim T., Zhang W., Hartmann N.B., Malpei F., Candelario V.M. (2022). Reuse of Water in Laundry Applications with Microand Ultrafiltration Ceramic Membrane. Membranes.

[51] Palacios-Mateo C., van der Meer Y., Seide G. (2021). Analysis of the polyester clothing value chain to identify key intervention points for sustainability. Environ. Sci. Eur..

[52] Cai Y., Mitrano D.M., Heuberger M., Hufenus R., Nowack B. (2020). The origin of microplastic fiber in polyester textiles: The textile production process matters. J. Clean. Prod..

[53] Carmichael A. (2015). Man-made fibers continue to grow. Text. World.

[54] Weber A., Scherer C., Brennholt N., Reifferscheid G., Wagner M. (2018). PET microplastics do not negatively affect the survival, development, metabolism and feeding activity of the freshwater invertebrate Gammarus pule. Environ. Pollut..

[55] Piccardo M., Provenza F., Grazioli E., Cavallo A., Terlitti A., Renzi M. (2020). PET microplastics toxicity on marine key species is influenced by pH, particle size and food variations. Sci. Total Environ..

[56] Čorak I., Pušić T., Tarbuk A. (2019). Enzimi za hidrolizu poliestera. Tekstil.

[57] Kärkkäinen N., Sillanpää M. (2021). Quantification of different microplastic fibres discharged from textiles in machine wash and tumble drying. Environ. Sci. Pollut. Res..

[58] Cesa F.S., Turra A., Checon H.H., Leonardi B., Baruque-Ramos J. (2020). Laundering and textile parameters influence fibers release in household washings. Environ. Pollut..

[59] Yang L., Qiao F., Lei K., Li H., Kang Y., Cui S., An L. (2019). Microfiber release from different fabrics during washing. Environ. Pollut..

[60] Carney Almroth B., Åström L., Roslund S., Petersson H., Johansson M., Persson N. (2018). Quantifying shedding of synthetic fibers from textiles; a source of microplastics released into the environment. Environ. Sci. Pollut. Res..

[61] Hartline N.L., Bruce N.J., Karba S.N., Ruff E.O., Sonar S.U., Holden P.A. (2016). Microfiber masses recovered from conventional machine washing of new or aged garments. Environ. Sci. Technol..

[62] Napper I.E., Thompson R.C. (2016). Release of synthetic microplastic plastic fibres from domestic washing machines: Effects of fabric type and washing conditions. Mar. Pollut. Bull..

[63] Corami F., Rosso B., Bravo B., Gambaro A., Barbante C. (2020). A novel method for purification, quantitative analysis and characterization of microplastic fibers using Micro-FTIR. Chemosphere.

[64] Belzagui F., Crespi M., Álvarez A., Gutiérrez-Bouzán C., Vilaseca M. (2019). Microplastics’ emissions: Microfibers’ detachment from textile garments. Environ. Pollut..

[65] Soljačić I., Pušić T. (2005). Njega Tekstila, Dio 1: Čišćenje u Vodenim Medijima, Zagreb, Tekstilno-Tehnološki Fakultet.

[66] Sinner H. (1960). Ueber das Waschen mit Haushaltwaschmaschinen: In Welchem Umfange Erleichtern Haushaltwaschmachinen und—Geraete das Waeschewaschen im Haushalt?.

[67] Bao W., Gong R.H., Ding X., Xue Y., Li P., Fan W. (2017). Optimizing a laundering program for textiles in a front-loading washing machine and saving energy. J. Clean. Prod..

[68] Konstadinos Abeliotis K., Amberg C., Candan C., Ferri A., Osset M., Owens J., Stamminger R. (2015). Trends in laundry by 2030. Househ. Pers. Care Today.

[69] Alfieri F., Cordella M., Stamiminger R., Bues A. Durability Assessment of Products: Analysis and Testing of Washing Machines, JCR Technical Reports. https://publications.jrc.ec.europa.eu/repository/bitstream/JRC114329/jrc114329_task_3_durability_final_v3.0.pdf.

[70] Kelly M.R., Lant N.J., Kurr M., Burgess J.G. (2019). Importance of water-volume on the release of microplastic fibers from laundry. Environ. Sci. Technol..

[71] O’Brien S., Okoffo E.D., O’Brien J.W., Ribeiro F., Wang X., Wright S.L., Samanipour S., Rauert C., Alajo Toapanta T.Y., Albarracin R. (2020). Airborne emissions of microplastic fibres from domestic laundry dryers. Sci. Total Environ..

[72] Kapp K.J., Miller R.Z. (2020). Electric clothes dryers: An underestimated source of microfiber pollution. PLoS ONE.

[73] Danyang T., Zhang K., Xu S., Lin H., Liu Y., Kang J., Yim T., Giesy J.P., Leung K.M.Y. (2022). Microfibers Released into the Air from a Household Tumble Dryer. Environ. Sci. Technol. Lett..

[74] Pirc U., Vidmar M., Mozer A., Kržan A. (2016). Emissions of Microplastic Fibers from Microfiber Fleece during Domestic Washing. Environ. Sci. Pollut. Res..

[75] Zhang Q., Xu E.G., Li J., Chen Q., Ma L., Zeng E.Y., Shi H. (2020). A Review of Microplastics in Table Salt, Drinking Water, and Air: Direct Human Exposure. Environ. Sci. Technol..

[76] Abbasi S., Keshavarzi B., Moore F., Turner A., Kelly F.J., Dominguez A.O., Jaafarzadeh N. (2019). Distribution and potential health impacts of microplastics and microrubbers in air and street dusts from Asaluyeh County, Iran. Environ. Pollut..

[77] De Falco F., Gullo M.P., Gentile G., Di Pace E., Cocca M., Gelabert L., Brouta-Agnésa M., Rovira A., Escudero R., Villalba R. (2018). Evaluation of microplastic release caused by textile washing processes of synthetic fabrics. Environ. Pollut..

[78] Zambrano M.C., Pawlak J.J., Daystar J., Ankeny M., Cheng J.J., Venditti R.A. (2019). Microfibers generated from the laundering of cotton, rayon and polyester based fabrics and their aquatic biodegradation. Mar. Pollut. Bull..

[79] Browne M.A., Crump P., Niven S.J., Teuten E., Tonkin A., Galloway T., Thompson R. (2011). Accumulation of microplastic on shorelines woldwide: Sources and sinks. Environ. Sci. Technol..

[80] Vassilenko E., Watkins M., Chastain S., Mertens J., Posacka A.M., Patankar S., Ross P.S. (2020). Domestic laundry and microfiber pollution: Exploring fiber shedding from consumer apparel textiles. PLoS ONE.

[81] Guo Q.Q., Xiao M.R., Ma Y., Niu H., Zhang G.S. (2021). Polyester microfiber and natural organic matter impact microbial communities, carbon-degraded enzymes, and carbon accumulation in a clayey soil. J. Hazard. Mater..

[82] Talvitie J., Mikola A., Koistinen A., Setälä O. (2017). Solutions to microplastic pollution—Removal of microplastics from wastewater effluent with advanced wastewater treatment technologies. Water Res..

[83] Sol D., Laca A., Laca A., Diaz M. (2021). Microplastics in Wastewater and Drinking Water Treatment Plants: Occurrence and Removal of Microfibres. Appl. Sci..

[84] Singh S., Madhanraj K., Vishal D. (2021). Removal of microplastics from wastewater: Available techniques and way forward. Water Sci. Technol..

[85] Menéndez-Manjón A., Martínez-Díez R., Sol D., Laca A., Laca A., Rancaño A., Díaz M. (2022). Long-Term Occurrence and Fate of Microplastics in WWTPs: A Case Study in Southwest Europe. Appl. Sci..

[86] Liu F., Nord N.B., Baster K., Vollertsen J. (2020). Microplastics removal from treated wastewater by a biofilter. Water.

[87] Zhang Y., Wang H., Xu J., Lu M., Wang Z., Zhang Y. (2021). Occurrence and Characteristics of Microplastics in a Wastewater Treatment Plant. Bull. Environ. Contam. Toxicol..

[88] Ramasamy R., Subramanian R.B. (2021). Synthetic textile and microfiber pollution: A review on mitigation strategies. Environ. Sci. Pollut. Res..

[89] Anis A., Classon S. Analysis of Microplastic Prevention Methods from Synthetic Textiles. iGEM LUND 2017. http://2017.igem.org/wiki/images/2/2c/T--Lund---Analysis_of_Microplastic_Prevention_Methods_from_Synthetic_Textiles.pdf.

[90] De Falco F., Gentile G., Avolio R., Errico M.E., Di Pace E., Ambrogi V., Avella M., Cocca M. (2018). Pectin based finishing to mitigate the impact of microplastics released by polyamide fabrics. Carbohydr. Polym..

[91] Bhavsar P.S., Fontana D.G., Zoccola M. (2021). Sustainable Superheated Water Hydrolysis of Black Soldier Fly Exuviae for Chitin Extraction and Use of the Obtained Chitosan in the Textile Field. ACS Omega.

[92] Matsukawa S., Kasai M., Mizuta Y. (1995). Modification of Polyester Fabrics Using Chitosan. Sen’i Gakkaishi.

[93] Flinčec Grgac S., Tarbuk A., Dekanić T., Sujka W., Draczyński Z. (2020). The Chitosan Implementation into Cotton and Polyester/Cotton Blend Fabrics. Materials.

[94] Korica M., Peršin Z., Trifunović S., Mihajlovski K., Nikolić T., Maletić S., Fras Zemljič L., Kostić M.M. (2019). Influence of Different Pretreatments on the Antibacterial Properties of Chitosan Functionalized Viscose Fabric: TEMPO Oxidation and Coating with TEMPO Oxidized Cellulose Nanofibrils. Materials.

[95] Zhang Z.-T., Chen L., Ji J.-M., Huang Y.-L., Chen D.-H. (2003). Antibacterial Properties of Cotton Fabrics Treated with Chitosan. Text. Res. J..

[96] Yilmaz Atay H., Jana S. (2020). Antibacterial activity of chitosan-based systems. Functional Chitosan: Drug Delivery and Biomedical Applications.

[97] Ortega-Ortiz H., Gutiérrez-Rodríguez B., Cadenas-Pliego G., Jimenez L.I. (2010). Antibacterial Activity of Chitosan and the Interpolyelectrolyte Complexes of Poly(acrylic acid)-Chitosan. Braz. Arch. Biol. Technol..

[98] Walawska A., Filipowska B., Rybicki E. (2003). Dyeing Polyester and Cotton-Polyester Fabrics by Means of Direct Dyestuffs after Chitosan Treatment. Fibers Text. East. Eur..

[99] Najafzadeh N., Habibi S., Ghasri M.A. (2018). Dyeing of Polyester with Reactive Dyestuffs Using Nano-Chitosan. J. Eng. Fibers Fabr..

[100] Dey T.K., Md U., Mamun J. (2021). Detection and removal of microplastics in wastewater: Evolution and impact. Environ. Sci. Pollut. Res..

[101] Gong J., Kong T., Li Y., Li Q., Li Z., Zhang J. (2018). Biodegradation of Microplastic Derived from Poly(ethylene terephthalate) with Bacterial Whole-Cell Biocatalysts. Polymers.

